# Adaptive Echolocation and Flight Behaviors in Bats Can Inspire Technology Innovations for Sonar Tracking and Interception

**DOI:** 10.3390/s20102958

**Published:** 2020-05-23

**Authors:** Clarice Anna Diebold, Angeles Salles, Cynthia F. Moss

**Affiliations:** Department of Psychological and Brain Sciences, Johns Hopkins University, Baltimore, MD 21287, USA; clarice.diebold@jhu.edu (C.A.D.); ANGIESALLES@jhu.edu (A.S.)

**Keywords:** biosonar, predictive tracking, tracking algorithms

## Abstract

Target tracking and interception in a dynamic world proves to be a fundamental challenge faced by both animals and artificial systems. To track moving objects under natural conditions, agents must employ strategies to mitigate interference and conditions of uncertainty. Animal studies of prey tracking and capture reveal biological solutions, which can inspire new technologies, particularly for operations in complex and noisy environments. By reviewing research on target tracking and interception by echolocating bats, we aim to highlight biological solutions that could inform new approaches to artificial sonar tracking and navigation systems. Most bat species use wideband echolocation signals to navigate dense forests and hunt for evasive insects in the dark. Importantly, bats exhibit rapid adaptations in flight trajectory, sonar beam aim, and echolocation signal design, which appear to be key to the success of these animals in a variety of tasks. The rich suite of adaptive behaviors of echolocating bats could be leveraged in new sonar tracking technologies by implementing dynamic sensorimotor feedback control of wideband sonar signal design, head, and ear movements.

## 1. Introduction

Tracking moving targets in noisy and complex environments is a challenge that must be solved by biological organisms and artificial systems alike. Autonomous machines, such as self-driving cars or motorized wheelchairs, make use of iterative algorithms to navigate and map new environments [1]. Sonar offers valuable advantages for environmental mapping and target tracking, particularly in dark environments, and biological solutions can inspire innovation in this technology arena [2].

Diverse animal groups have evolved strategies for tracking moving targets by generating estimates of target motion. Much of the biological research that informs current understanding of target tracking in animals focuses on visually dominant species. Some organisms use a constant target-bearing strategy, such as linear optical trajectory (LOT) strategy, to maintain a fixed relationship between heading angle and a selected target, to eventually intercept a prey item [3,4], while other organisms use predictive internal models to anticipate the motion of erratically moving prey [5,6]. Biological models have served to inspire optimization and tracking algorithms, including cuckoo birds [7], ants [8], and fireflies [9]. Auditory-specialists, like the echolocating bat, provide a powerful biological model for target tracking by sonar. Bats are the only mammals capable of powered flight [10] and can dynamically modify both path planning and echolocation signal design as they track and approach target [11,12]. They also display differences in flight and echolocation behaviors in open and cluttered environments [13]. The rich suite of adaptive behaviors exhibited by echolocating bats operating in different environments can serve to inspire technological advances in sonar tracking and localization algorithms. 

Here, we review the bat’s dynamic sonar and flight behaviors as they perform natural tasks, with a focus on tracking and pursuit strategies across ecological niches. Our goal is to highlight the dazzling display of bat adaptive behaviors, which engineers could implement in new technological applications and innovations. 

## 2. Echolocation in Bats

Over 1000 species of bats echolocate [14]. The majority of echolocating bats produce signals with the larynx, emitting ultrasonic calls through the mouth or nose. There are some exceptions, such as *Rousettus aegyptiacus*, which emits ultrasonic clicks with the tongue. The discrete sonar signals emitted by echolocating bats reflect from objects in the path of the sound beam and return to the bat in the form of echoes. Laryngeal echolocating bats can emit pulses as short as 0.5 milliseconds, with frequencies that typically range from 25 to 150 kHz, though some bats produce sonar calls at frequencies outside that range [15,16,17]. Bats use the features of returning echoes to generate 3D representations of their surroundings [18,19,20].

**Figure 1:**
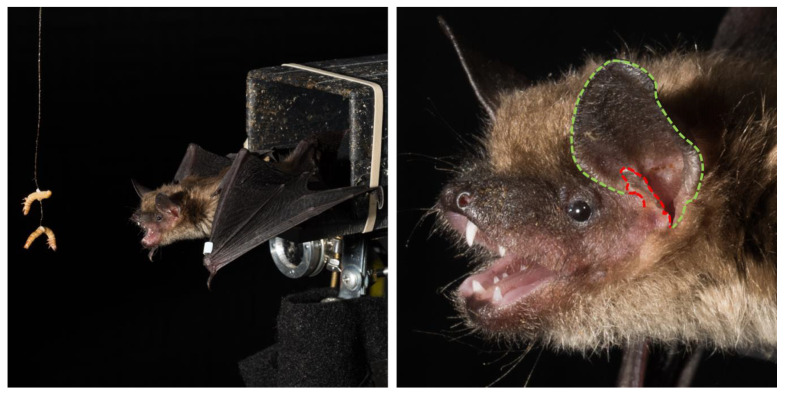
*Eptesicus fuscus* bat. Left panel: Bats are trained to perch on a platform and produce echolocation calls to track and intercept approaching targets (mealworms). This experimental setup allows us to study bat sonar tracking behavior while maintaining careful control of the target motion. Right panel: Closeup of the head of the bat, showing details of the external ear anatomy. A green dashed line delineates the left pinna, which acts as a receiver and can be independently moved to control inter-aural differences, necessary for azimuthal localization of targets [24]. The red dashed line delineates the enlarged tragus, which contributes to target elevation estimation. Photos courtesy of Dr. Brock Fenton.

The anatomical structure of the bat’s outer ears functions as two receivers with a specialized skin flap, known as the tragus (see Figure 1). The tragus introduces elevation-dependent spectral changes in echoes, which bats can use for vertical localization [21,22]. Inter-aural differences are used by the bat to estimate the horizontal location of objects with accuracy of ~1.5 deg [23]. Bats can enhance cues for sound localization by moving their head and pinna independently, to amplify interaural differences used to localize sonar targets [24,25]. Finally, bats rely on the time delay between each sonar call and echo return to gauge the distance to a target, showing distance-difference discrimination thresholds of approximately 1 to 3 cm [18,26], depending on the species. Importantly, bats dynamically modify the spectro-temporal features of sonar calls with respect to task (e.g., search, approach, and interception phases of foraging) and the environment (e.g., dense vegetation or open space) [27]. These adaptations rely on a robust audio-motor feedback system that supports advanced navigation and tracking behaviors.

Different species of bats have evolved specialized sonar signal designs. Call types can broadly be broken into two different categories: frequency modulated (FM) signals and constant frequency (CF) signals. FM signals sweep across a broad range of frequencies and are well suited for target localization, whereas CF signals are narrowband tones that are typically longer in duration than FM signals, and they tend to be used by bats that hunt for fluttering targets in dense vegetation [17,28,29]. CF sonar signals are often combined with FM components (CF-FM), whose bandwidth increases when animals must estimate target distance [18]. Sonar call structures depend on the environment and preferred prey of a bat. FM sweeps alone are employed by most echolocating bats and can vary in bandwidth, according to the task at hand. FM bats that forage in open fields tend to emit narrowband FM search calls with low duty-cycle, to detect prey, and shift to broadband FM signals to intercept and capture evasive insects. Bats that forage in or near clutter emit short, very broadband FM calls, to reduce masking effects by the echoes returning from nearby foliage [30]. CF–FM bats rely on Doppler Shift Compensation (DSC), compensating for the Doppler shift introduced by their own movement by lowering the frequency of emitted calls to stabilize the frequency of returning echoes to a band that they hear best (i.e., detection and frequency discrimination thresholds are lowest) [31,32,33].

While the call structures described above can aid in tracking targets in cluttered conditions, bats still must contend with masking effects when target echoes are obscured by other sounds. Forward masking occurs when the interfering signals precede the target signal, backward masking occurs when the interfering signals follow the target signal, and simultaneous masking occurs when interfering signals return at the same time as the target signal [17]. To reduce interference from signals in their environment, bats may adjust the duration of their sonar emissions, to reduce overlap of target echoes with their own echolocation broadcasts and clutter echoes [34]. Some species of bats avoid dense clutter conditions altogether [35]. In laryngeal FM echolocators, echoes that return from objects off-axis from the sonar beam axis are weaker and low-pass filtered, allowing the bat to separate clutter echoes from on-axis target echoes [36]. In conditions with multiple objects that return a cascade of echoes for each sonar emission, bats may change flight velocity and path planning, to reduce clutter interference [13,37].

Echolocating bats show additional adaptive sonar behaviors to track objects and avoid obstacles. For example, bats adjust the directional aim of sonar signals to detect and localize objects in the environment [38,39,40]. Some bats alternate between emitting sounds in groups at short inter-pulse intervals (20–40 ms) and longer inter-pulse intervals (>50 ms) in cluttered environments [37,41,42,43]. They may also make frequency adjustments in successive echolocation calls, possibly to facilitate pulse-echo assignment when multiple echoes return at different delays from clutter objects extended along the range axis [44].

Along with acoustic interference in reverberant, cluttered habitats, bats must also operate in a cocktail-party-like environment, where they must parse echoes from their own calls and the sonar signals from other bats, to select and track sonar targets, while also listening in on social calls produced by nearby conspecifics [11,45,46]. In acoustically complex environments, bats employ a vast array of behavioral strategies to maximize target information and minimize interference [47]. In the presence of conspecifics, bats may adjust frequencies of signals or cease calling entirely, to reduce sonar jamming [46], or some species, such as *Tadarida brasiliensis*, produce sinusoidal FM calls to jam the echolocation of competing bats for food [48]. Bats have also been shown to eavesdrop on the sounds produced by bats foraging nearby [49]. The ability to quickly modify behavior to counter masking and potential jamming signals is a key adaptation bats exhibit, to minimize signal interference.

Bats are auditory specialists that have evolved a high-resolution active sensing system to represent objects in their surroundings, for the purpose of target tracking and obstacle avoidance. The adaptations of bats from their engagement in natural tasks have inspired sonar technology, but the *full suite of strategies used by bats remains to be exploited in the advance of artificial sonar systems*. 

## 3. Target Tracking by Echolocating Bats

Many predatory bats track moving insect prey while navigating through cluttered environments. This creates an added cognitive challenge: Not only must the bats use intermittent echo returns from stationary objects, such as foliage and buildings, to steer around obstacles, but they must also process echoes from moving prey items to track target trajectories and plan successful interception. As described above, bats use the time delay between each call and echo to estimate target range [18]. However, when tracking prey, the bat’s estimate of a moving target’s position is obsolete by the time the bat receives information carried by the most recent echo. Delays accumulate from the time it takes for (1) a sonar broadcast to travel to the object, (2) the echo to return to the bat’s ears, (3) the brain to process information from the returning echo, and (4) the generation of an appropriate motor response. These delays collectively can add up to as much as 100 ms following each sonar transmission [27]. To accommodate these delays, bats have evolved sophisticated tracking strategies, adapted both to movement patterns of prey and features of the environment. 

### 3.1. Sonar Tracking Behaviors

**Figure 2:**
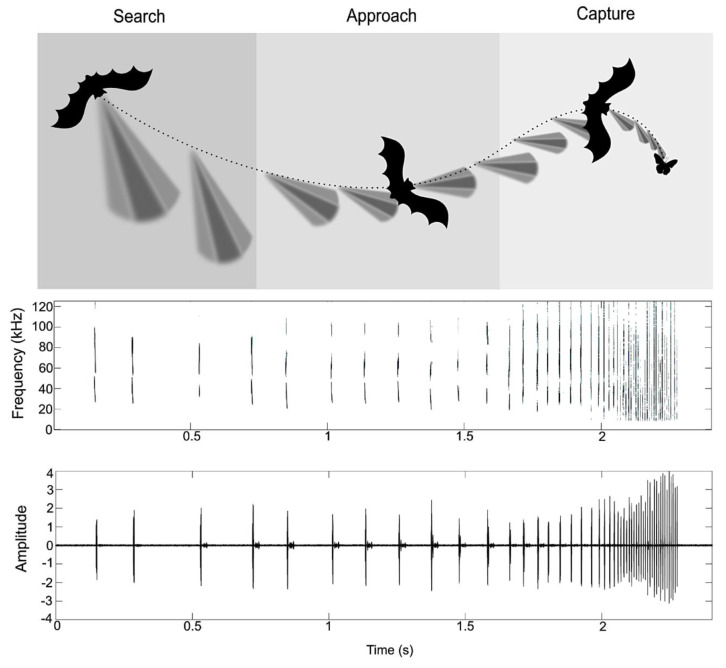
Classic insect-pursuit sequence of an FM bat. The top panel shows a depiction of a bat pursuing an insect. The grayscale fans illustrate the directional aim of the bat’s sonar beam, with the darkest regions illustrating the beam axis containing greatest sound energy. In the search phase, bats orient their beam aim to scan the environment in different directions and emit narrowband, long-duration sonar calls. The approach phase commences when echo information about a target returns to the bat; it is characterized by an increase in FM bandwidth, the bat locking its sonar beam aim onto the selected target, and the bat increasing its rate of sonar calls. Finally, when the bat moves to capture the insect, it emits a quick succession of calls, further decreasing the inter-pulse interval, until it intercepts the target. The center panel depicts spectrograms (time frequency representations) of natural echolocation calls from a target tracking sequence of a big brown bat, *Eptesicus fuscus*, in the lab, and shows the approach and capture phases of insect pursuit (low signal-to-noise ratio may have affected the quality of the spectrograms of some signals). The lower panel shows the waveform of the above sequence. Increases in signal amplitude with decreasing target distance are an artifact of the recording conditions. These panels illustrate the change in sonar-call repetition rate in a bat approaching and intercepting a target.

Sonar tracking strategies in FM-calling aerial hawking insectivores like *Eptesicus fuscus* reveal the fast-dynamic modifications in sonar behavior as the bat approaches a target. In open environments, bats emit long (8–25 ms), low frequency (<30 kHz), narrowband search signals. The shallow FM search signals are produced at a low repetition rate, as infrequently as every-other wingbeat (interpulse intervals ~200 ms). Approach calls are usually broadband signals (duration 2–6 ms), sweeping over 30–120 kHz. As FM bats approach a target, they lock their sonar beam on the prey item and reduce their signal duration and pulse intervals, until they prepare to intercept their target by emitting 0.5–1 ms signals at a high repetition rate (150–200 Hz) [17,50,51]. A similar trend seen in CF–FM bats, with the duration of the CF component and the bandwidth of the FM component of calls modified as the animal approaches a target [52,53]. Environmental conditions, clutter, and prey identity all contribute to further specializations of this insect-pursuit sequence (Figure 2).

### 3.2. Tracking Evasive Prey

When targets move in linear trajectories, many different organisms, including falcons [54], dogs [3], and fish [55], track moving targets by approaching along a straight trajectory, while keeping constant the angle between the animal’s heading and the selected target, as the distance between the two decreases. This strategy is known as a constant bearing (CB) strategy, which is effective for intercepting a target moving along a straight and predictable path. However, the pursuer of an erratically moving target would never converge to the optimum bearing by using the CB strategy.

Many insectivorous bats must contend with prey that can actively maneuver to avoid capture and even jam echolocation. The predator–prey dynamics between bats and insects have revealed an evolutionary arms race that produces extremely specialized behaviors through selective pressures. Many different insects have evolved hearing sensitivities in the ultrasound frequency ranges of the echolocation signals used by predatory bats [56,57,58,59]. Some insects have also evolved various evasive flight maneuvers in response to bat signals, from highly stereotyped linear movement away from the bat, demonstrated by Coleopterans (beetles) [56,60,61], to erratic flight trajectories in Lepidopterans (butterflies and moths). Praying mantids have a cyclopean ear to detect bat ultrasound and drop to the ground in response to sonar signals [56]; lacewing moths also cease flying and suddenly plummet downward when they detect the hunting echolocation calls of their main predator, *Pipistrellus pipistrellus*, [57]. This plummeting strategy significantly decreases capture success by the bats [62,63,64]. Additionally, some insects, such as the tiger moth *Bertholdia trigona,* have developed ultrasonic clicks, which disrupt the bat’s ability to successfully track prey by using echolocation [65]. When bats hear tiger moth ultrasonic clicks, they reverse their insect-capture-sequence pattern by elongating call durations and pulse intervals, likely to contend with multiple sound streams [66]. Bats must not only employ their own tracking strategies for capturing moving targets in midair, but also contend with counter strategies that insects have developed specifically to evade capture.

**Figure 3:**
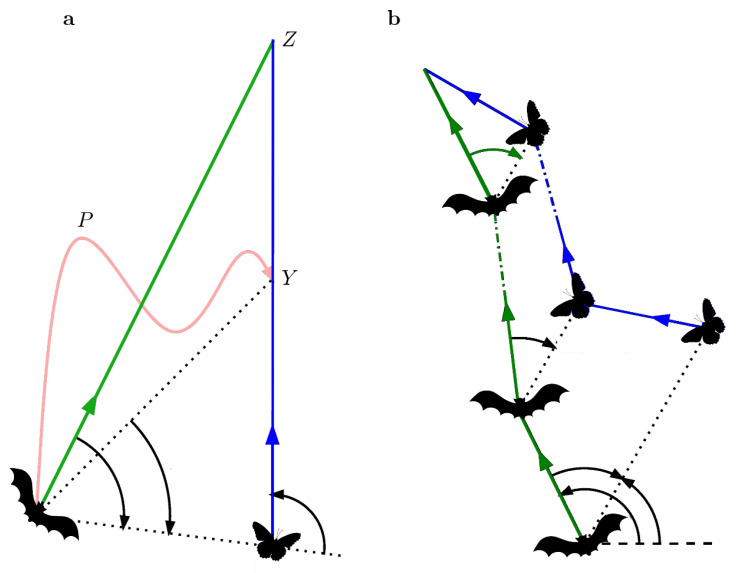
Modified from Ghose et al. 2006 [67]. (**a**) Constant bearing strategy (CB). Target (insect) moves in a straight line, at a constant velocity (blue line), and is pursued by bat that holds a fixed target bearing (green line), aiming where it will intercept the target (interception point Z). An alternative nonlinear path (pink) can be adopted by the bat when pursuing a target with constant linear velocity, resulting in a shorter intercept time at point Y (see Ghose et al. 2006 for further description). (**b**) Constant Absolute Target Direction strategy (CATD). Target (insect) moves erratically, by changing both direction and speed along path (blue). True erratic target motion cannot have a global time-minimum intercept, but can be approximated by infinite constant velocity segments. The bat’s path (green) can follow a locally time-optimal path by adjusting its flight trajectory to minimize changes in the absolute direction of the target. This strategy relies on the target position update acquired from returning echoes.

The challenges echolocating bats face in capturing erratically flying insect prey means that a CB strategy would not incorporate the flexibility needed for successful capture. By extension, it has been proposed that bats maintain an optimal bearing by minimizing changes in the absolute direction relative to the target, termed a constant absolute target direction (CATD) strategy [67]. The CATD model posits that an animal generates and updates internal estimations of the distance and direction of the target relative to itself (in the bat through echolocation), to compute a time-optimal strategy for intercepting erratically moving targets (Figure 3). The CATD strategy, analogous to parallel navigation [68], has been demonstrated in predatory robber flies [69] and interpreted as a strategy for motion camouflage in dragonflies [70]. It has also been implemented in models with a sensorimotor feedback system that relies on delays, which may have application for unmanned vehicle control [71].

Although the CATD strategy suggests that bats build an internal model of target motion, the echolocating bat’s implementation of predictive strategies for target tracking has been a controversial topic. Masters and colleagues previously reported that the big brown bat *Eptesicus fuscus* uses a non-predictive strategy when tracking a moving target, orienting head aim to the location of the last returning echo, rather than the target’s actual location [72]. Further studies in bats, however, indicate that a non-predictive model cannot account for the success of bats tracking occluded or evasive targets. Behavioral studies of the fishing bat *Noctilio leporinus* demonstrated that this species could use the movement of an artificial fish before it disappeared under water from the acoustic view of the bat, to accurately plan target interception [73]. Computational modeling of the trajectories of foraging bats have shown that anticipatory motor planning reflects realistic capture performance [74], and that bats attend to the future location of prey items in a sequence, to guide flight-path selection and improve capture rates [75]. Recently, we conclusively demonstrated that *E. fuscus* relies on predictive models of target trajectories when tracking moving targets [76]. Specifically, we have empirical evidence to refute the Masters et al. [72] claim of non-predictive tracking, and show that bats rely on a predictive model to track moving objects and even continue to track targets when echoes are blocked by an occluder during a portion of the target’s trajectory. This suggests a strategy bats may employ to contend with insect prey that disappear momentarily behind clutter in the environment. Furthermore, we found that when internal models of target motion were violated by unpredictable changes in velocity, bats quickly modified echolocation behavior, to probe the environment for additional information, in order to update internal models and resume tracking the target. Our behavioral data align with a model in which bats estimate target velocity based on the echo arrival time differences between past sampled locations and further advance head aim by a fixed angle. Bats are able to track evasive or occluded targets by building predictive models of target trajectory and use this information to successfully intercept erratic prey. Future studies will investigate constraints on sonar tracking models that bats use to navigate in complex environments.

## 4. Adaptive Bat Echolocation Behaviors Inspire Artificial Sonar Tracking Systems

The echolocation and flight behavior of bats have been a source of inspiration for many technological advances, however, many key features of bat sonar tracking have yet to be realized in artificial systems. As described above, bats rapidly modify the spectro-temporal features of echolocation calls, and these adaptive sonar behaviors are fundamental to their performance in navigating complex environments while tracking and intercepting targets. Full implementation of these adaptive sonar behaviors, coupled with the use of wideband sonar signals, offers great potential for future technology applications. In this section, we present some examples in which bats have inspired technology thus far.

In 2010, a standard bat algorithm (BA) was proposed as a metaheuristic algorithm that uses similar processes of echolocating bats for global optimization [77]. The standard BA uses idealized behaviors of echolocating bats, which draws from a limited subset of parameters. These idealized behaviors or rules are as follows:

1. Bats use echolocation to sense distance and can identify and categorize targets relative to background barriers.
2. Bats fly randomly, varying the frequency and intensity of narrowband echolocation signals to detect prey, and can adjust the parameters of their sonar sounds relative to their distance to the target.
3. Call intensity varies, from a large positive value to a minimum constant value.

This algorithm iteratively updates the position and velocity of a virtual bat, using these three idealized rules. This updating allows for a more dynamic and efficient method for optimizing the processing of sensory information, allowing the BA to solve constrained and unconstrained optimization problems better than similar biologically inspired algorithms [78,79]. However, these idealized rules greatly simplify the components of adaptive echolocation, e.g., assuming that bats use a single sonar frequency, which changes with distance. The algorithm does not consider the bat’s use of wideband FM signals or task-dependent behavioral measures at a given distance, such as preparing to intercept a target vs. flying by that target. The standard BA has been further developed to incorporate a directional bat algorithm (dBA), which improves performance in different types of environments and conditions, including premature convergence due to low exploration [80]. More recent advances in a binary bat algorithm (bBA) address traffic network determination problems [81] and parameter initialization to improve convergence velocity and accuracy [82]. Further integration of a more complete repertoire of adaptive behaviors of bats would continue to improve this optimization algorithm. 

Robotic navigation has employed the Simultaneous Localization and Mapping (SLAM), which constructs and updates a spatial map of a novel, fixed environment, from both allocentric and egocentric perspectives, thus allowing an agent to navigate without *a priori* knowledge of the surroundings. In the last decade, there have been significant strides in creating reliable SLAM algorithms, however, there are still limitations to these approaches. Sensor uncertainty, the processing demands of complex computations, and challenges of dynamic environments contribute to the current limitations of SLAM algorithms [83]. One approach to this problem is RatSLAM, which uses the computational models of the hippocampus in rodents to inform navigation in novel environments with ambiguous landmark information [84]. This biologically inspired approach to SLAM has yielded promising results, with increased place-recognition performance and recovery from path integration errors. Expanding on this biological model, Steckel and Peremans have proposed the use of the echolocating bat for a sonar-based model of SLAM [2], which can operate under conditions where optical information is reduced or unavailable. Previous SLAM systems with sonar capabilities required impractically large numbers of sonar measurement to converge on a functional map [2], but BatSLAM offers a new way to use sonar information more efficiently, to allow autonomous sonar-guided robots to navigate an environment. Like Yang’s Bat Algorithm, BatSLAM draws inspiration from the bat’s biosonar, to localize the positions of obstacles to generate an experience map and modify motor commands for path integration. Additionally, they use the physical structure of the bat’s external ears to allow binaural sound localization, though they do not incorporate adaptive sonar signal design, head movements, or the ability of the bat to dynamically move each pinna independently, to amplify interaural differences (Figure 1). Combining directionality of sonar emissions and binaural echo reception of bats, Steckel and Peremans developed the Echolocation Related Transfer Function (ERTF) for spectro-spatial filtering, realizing a biomimetic sonar system that localizes multiple acoustic objects with wideband sonar [2]. It creates consistent maps of large environments that can converge over time, to relatively accurate metric maps, to support navigation. More recently, there have been advances to BatSLAM, which include odometric information and an acoustic flow model, which allows for a novel 3D sonar sensor [85], as well new optimization of 2D-experience mapping through the addition of an audio-aware perceptual hash with a closed-loop detection algorithm, using fixed CF and FM sonar signals [86]. These new enhancements to BatSLAM enable richer representations of complex environments, however, dynamic bat sonar adjustments offer many additional features that could be incorporated in future versions, for operation in more complex and dynamic environments. 

Tracking algorithms have a myriad of uses, from surveillance [87] to biomedical applications [88]. While the accuracy of tracking algorithms has improved significantly in the last decade, they often fail to contend with background noise and clutter, which interferes with localization of a selected target. Kalman filters operate with iterative processes that aid in estimating the position and motion of a target and have been a standard for addressing the challenge of noisy target data. The addition of Kalman filters to tracking algorithms dramatically improves tracking fidelity and reduces interference by local maxima [89], particularly in linear systems. In nonlinear systems, extended Kalman filters also perform an iterative process with increased success [90], but concerns have been raised about inconsistent mapping and a penchant for underestimating covariance [91], particularly in handling sonar and vision data for the bearing of a target [92,93]. Improvements to complex tracking-condition algorithms have proven promising, such as application of multiple Kalman filters, which allows precise tracking of dynamically moving targets [94]. However, target tracking by artificial systems remains a challenge, and target interception success is still low.

**Figure 4:**
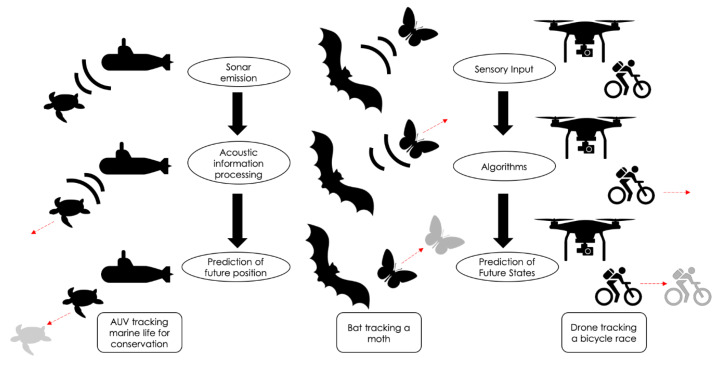
Bats as a biological model to inspire tracking algorithms. Tracking moving targets requires sensory information that can be in the form of echoes (left/center panels) or visual stimuli (right panel). This information must then be processed by computations that allow for the prediction of future states (shown in gray). Man-made systems like Autonomous Underwater Vehicles (AUV) use sonar to track different moving targets, such as marine life and wildlife (left panel). Bats acquire discrete sensory information in the form of echo returns from adaptive sonar emissions; echo snapshots are integrated, to enable the prediction of the future position of a moving insect (center panel). These predictive tracking algorithms can be implemented in technologies that use sonar or other sensory modalities, such as drone videography of a bicycle race (right panel).

Finally, biologically inspired approaches to sonar tracking algorithms have hailed some success, with iterative predictive algorithms approaching performance levels comparable to biological systems [95]. Both for biological and artificial systems, real-world tracking requires sensory input, which is then processed to output accurate pursuit of moving targets (Figure 4). Some models have implemented rudimentary behavioral features of bat flight trajectories and putative predictive tracking [74]. Behavioral research on bat sonar target tracking has provided valuable insights into the strategies these animals employ to perform complex tasks, including differential adaptation of their echolocation behavior with respect to moving targets and stationary obstacles [96], as well as Doppler shift compensation and discrimination [31], all while contending with different environmental constraints and conditions [27,30]. Empirical studies of adaptive and predictive sonar tracking behaviors of bats [76], in conjunction with neurophysiological experiments, will provide insights to the computations employed by echolocating animals to carry out tasks under real-world conditions, and in turn provide further inspiration for new algorithms and neural networks that will improve artificial tracking systems. 

## 5. Conclusions

Our review aims to highlight the richness of adaptive sonar behavior and performance exhibited by diverse bat species, which collectively can inspire exciting advances in sonar tracking technology. Bats rely on a highly developed audio-vocal feedback system that supports computation of the distance and direction of objects in their surroundings. Most bats make use of wideband sonar signals and dynamically modify the spectro-temporal features of echolocation sounds in response to sensory information about the location of targets and obstacles. Bats navigate highly complex environments, identify targets relative to surrounding clutter, and are able to anticipate target motion, in order to intercept and capture moving targets in flight. Bat echolocation has inspired sonar technology advances for decades, however, artificial systems have yet to incorporate the full richness of adaptive sonar behaviors for target tracking and interception.
